# Our Contemporaries

**Published:** 1916-11

**Authors:** 


### OUR CONTEMPORARIES

**Inadequacy of Privately-Feed Medicine.** Dr. Inez C. Philbrook, Western Medical Review, Sept. and Medical Record, Sept. 9, discusses the gradual disuse of clinical pathology, methods of specialists, as the practitioner becomes more busy in general medicine, and calls attention to the need of institutional care of many cases and to the advantages of salaried positions, with official recognition, leisure for research, etc. To a certain extent but in a very different spirit, Dr. Philbrook emphasizes the point recently made by Dr. Richard Cabot of Boston regarding hospital efficiency. In our experience, very few physicians are contented with official, salaried positions, unless of a kind much above the average standard of professional prestige and income. Some years ago, while temporarily holding a position for a friend who was on a vacation, it seemed that the conditions were ideal and the position was extremely well fed if not very highly feed beyond maintenance. Yet a physician permanently engaged in the same work longed for "a thousand dollar private practice in a little village in Ohio." Except in certain lines of laboratory work—and without even this exception for the pioneers—the same general rule applies to medicine as to any other profession or business, namely that both from the standpoint of the individual and of society, the most is accomplished by the man who is his own master. Sometimes it even seems that the best professional success is made by those who are interested in practice not only scientifically and philanthropically, but who depend on it for a livelihood.

---

Medical Liberty pays the following compliment to physicians: "Vote for an imbecile, a nigger, or a yaller dog in preference to a medical beast who would arm himself with the law (the coward's club) and when death hovers over the brow of your dying wife or child refuse you the right to employ assistance outside the pill and powder gang of which he is the putrescent tool and procurer."

---

The Illinois Medical Journal, Oct., very appropriately places next to its Automobile Dept. a report of the Ford Co. Medical Society.

---

The Century, Oct., publishes an anticipatory ghost story entitled the Burned House, by Vincent O'Sullivan. The narrator, marooned in a small village, takes an evening walk and sees a man approach a house stealthily. In a few minutes, the house bursts into flames and a man and a woman are seen cut off from help. He notes that, though standing near, he feels no heat from the flames. Returning, he finds the man first seen hanging from a bridge and pulls up his body, which is as light as paper. Not unreasonably, the narrator is doubtful of the reality of what he has seen and on making inquiries is not surprised to learn that there was not and never had been a house where he saw it before and during the fire. Several years later, he returns to the same village and learns that in the meantime a man had built a house on that location for his son who had occupied it with his bride and both burned to death, a disappointed suitor having set fire to the house and committed suicide as related. We have no objection to the publication of circumstantial evidence to support the belief in ghosts or any other supernatural phenomenon.

We will even go so far as to say that we are perfectly willing to believe in ghosts or anything else of the sort, if proper evidence is submitted to us. Neither have we any objection to fiction as such. But stories of this sort, told in a realistic way undoubtedly tend to maintain superstitious beliefs. Readers naturally forget just where they have heard or read them and the impression of truth remains and fosters a misconception which prevents popular enlightenment.

It may be said that we are taking fiction too seriously but here is an occurrence of October 5 that may be something more than a coincidence; Mr. and Mrs. Chas. Van Etten, a young couple of Estey's Point near Ithaca, were shot and seriously wounded early in the morning by Andrew Stinard, a neighbor, whom they discovered in their house with a large quantity of kerosene. Stinard is supposed to have become insane. It is pertinent to inquire if he had read the latest issue of the Century.

